# A magic kick for regeneration: role of mesenchymal stromal cell secretome in spermatogonial stem cell niche recovery

**DOI:** 10.1186/s13287-019-1479-3

**Published:** 2019-11-21

**Authors:** Georgy Sagaradze, Nataliya Basalova, Vladimir Kirpatovsky, Dmitry Ohobotov, Peter Nimiritsky, Olga Grigorieva, Vladimir Popov, Armais Kamalov, Vsevolod Tkachuk, Anastasia Efimenko

**Affiliations:** 1 Medical Research and Education Center, Lomonosov Moscow State University, Moscow, Russian Federation; 2 Faculty of Medicine, Lomonosov Moscow State University, Moscow, Russian Federation; 3 Research Institute of Urology and Interventional Radiology named N.A. Lopatkin - branch FSBI National Medical Research Radiological Center of the Ministry of Health of the Russian Federation, Moscow, Russian Federation

**Keywords:** Stem cell niche, Spermatogenesis, Mesenchymal stromal cells, Mesenchymal stem cells, Regeneration

## Abstract

**Background:**

Injury of stem cell niches may disturb tissue homeostasis and regeneration coordinated by specific niche components. Yet, the mechanisms of stem cell niche restoration remain poorly understood. Herein, we examined the role of mesenchymal stromal cells (MSCs) as pivotal regulators of stem cell niche recovery focusing on the effects of their secretome.

**Methods:**

The spermatogonial stem cell (SSC) niche was selected as a model. SSC niches were injured by inducing abdominal cryptorchidism in rats. Briefly, testes of anesthetized rats were elevated into the abdominal cavity through the inguinal canal for 14 days. After descent of testes, MSC or MSC secretome treatment was applied to the animals by local subtunical injections.

**Results:**

Local administration of MSC or MSC secretome was sufficient to recover spermatogenesis and production of functional germ cells. The effects of MSC and their secreted components were comparable, leading to restoration of Sertoli cell pools and recovery of Leydig cell secretory functions.

**Conclusion:**

Our data suggest that MSCs mimic the functions of lost supportive cells within the stem cell niche, transiently providing paracrine stimuli for target cells and triggering tissue regenerative processes after damage.

## Background

Adult stem cells in the microenvironments of stem cell niches are functional units of tissue homeostasis and regeneration. The niche is indispensable for stem cell function because it maintains stem cell pools and regulates cell behaviors in accordance with neighboring and distant cues [1, 2]. To participate in tissue renewal or restoration of injured tissue, resident quiescent stem cells must proliferate and differentiate into functional cells [3, 4]. Concomitantly, differentiation processes might depend on signals from stem cell niche components [5–7]. Thus, to guarantee sufficient tissue regeneration, coordinated niche restoration is a likely priority. However, the mechanisms regulating this process remain elusive.

Accumulating data indicates that mesenchymal stromal cells (MSCs) might be principal managers of stem cell niche regeneration after tissue injury. In particular, murine bone marrow responses of MSCs to signals that are associated with stimulation of niche regeneration lead to increased numbers of MSCs followed by expansions of stem cell populations [8, 9]. Additionally, after exposure to damage-associated stimuli, MSCs secrete a wide spectrum of growth factors, cytokines, and extracellular vesicles [10, 11], and some of them can at least contribute to the restoration of bone marrow as well as colon homeostasis [1, 12]. Furthermore, MSCs provide regulatory cues to stem cell niche components that also affect stem cell fates [13]. MSCs exert regenerative effects mostly by secretion of products that influence resident stem and progenitor cells and may regulate other niche cells too. However, other mechanisms including direct cell-to-cell communications, mitochondrial transfer, and differentiation may also be regarded [14–17]. Nevertheless, few studies consider the roles of MSC in stem cell niche regeneration.

The objective of this study was to examine the potential of MSCs to coordinate stem cell niche recovery by secreting paracrine factors. As a model for analysis, we selected the spermatogonial stem cell (SSC) niche. This niche represents an “open niche” microenvironment in which pools of self-renewing and differentiating cells are balanced. Other model niches contain other stem cell types that continue to divide asymmetrically after cessation of self-renewing signals [18]. This distinction is of great importance, because various interactions between stem cell niches and differentiating cells can be analyzed using this model.

In our study, the SSC niche in rats was injured by imposing bilateral abdominal cryptorchidism. The feasibility of this model to reproduce complex SSC niche failure as well as to evaluate the drug-driven regenerative effects on spermatogenesis restoration was shown previously [19]. Subsequently, MSC or MSC secretory products were locally injected and the regulatory impacts of MSC on SSC niche recovery were investigated. Established relationships indicate involvements of MSC in coordinated stem cell niche regeneration and provide proof of principle for applications of the MSC secretome in regenerative medicine.

## Methods

### Animals

The mature healthy male Wistar rats used in this study were of age between 3.5 and 4.0 months and had standard weight characteristics. Animals were housed and used for experimental procedures in full compliance with Directive 2010/63/EU.

### Manufacturing of human adipose MSC secretome

Samples of human adipose-derived MSC from the collection of the Cryobank of the Institute for Regenerative Medicine of Lomonosov Moscow State University (collection ID MSC_AD_MSU, www.human.depo.msu.ru) were used. MSCs were cultured in HyClone AdvanceSTEM cell culture media (GE Life Sciences, USA) containing 10% AdvanceSTEM Stem Cell Growth Supplement (GE Life Sciences, USA) and 100 U/ml penicillin/streptomycin (Gibco, USA). Immunophenotype of MSC was analyzed prior to adding cells to the collection (Additional file 1: Figure S1).

MSC-conditioned medium contained components of the MSC secretome (Additional file 2: Table S1, Additional file 3: Figure S2) and was obtained according to a previously established protocol [20]. Briefly, subconfluent MSCs at passages 4–5 were thoroughly washed with Hanks’ solution (PanEko, Russia) and were then cultured for 7 days in DMEM containing low glucose (DMEM-LG), GlutaMAX™ Supplement, pyruvate (DMEM-LG; Gibco, USA), and 100 U/ml penicillin/streptomycin. The aspirated MSC secretome was freed of cell debris by centrifugation for 10 min at 300*g* and was concentrated 25-fold using a centrifugal ultrafilter with 10 kDa molecular weight cutoff (MWCO; Merck, Germany).

### Abdominal cryptorchidism modeling

The technique for abdominal cryptorchidism modeling was described previously [19]. Briefly, testes of anesthetized rats were elevated into the abdominal cavity through the inguinal canal and fixed by the nodal suture to the abdominal wall in the region of the lateral canals with the atraumatic Prolene 4/0 for 14 days. To avoid possible blockage of connection between seminiferous tubules and epididymes, the distal pole of the testicle was sutured. After descent of testes, no treatment was applied to control rats (*n* = 6). Animals from the vehicle group (*n* = 8) were injected with a mixture of 50 μl of 2.5% bovine collagen gel (Imtek, Russia) and 50 μl of DMEM-LG. Subsequently, 250,000 MSCs in 100 μl of DMEM-LG medium were injected into rats of the MSC group (*n* = 8). Secretome group animals (*n* = 8) were treated with combination of 80 μl of 2.5% bovine collagen gel and 20 μl of 25-fold concentrated MSC secretome. All treated animals received local subtunical injections in a total volume of 100 μl right after descent of testes using an insulin syringe.

### Male rat fertility assessment

Abdominal cryptorchidism was modeled as described above. Additionally to previous groups, no treatment was applied to control rats (*n* = 8). Secretome group animals (*n* = 9) were treated with combination of 80 μl of 2.5% bovine collagen gel and 20 μl of 25-fold concentrated MSC secretome after descent of testes; 7 animals remained intact (unaltered control). Ten days before 3 months observation period, male rats were placed with females in proportion 1 to 2. Ten days after, percentages of pregnant female rats were calculated.

### Histological and immunohistochemical-fluorescence analyses

Both testicles were excised together 1 or 3 months after descent, were placed in buffered 10% neutral formalin solution for 24 h, and were then embedded in paraffin. Transverse 10-μm-thick sections of testicles were cut and placed on Polysine Adhesion Slides (Menzel, Germany). The sections were dewaxed and then conditioned in 0.01 M citrate buffer for 20 min at 98 °C to retrieve antigens. Sections were stained with DAPI (Sigma, Germany) and anti-PCNA antibodies (M0879, Dako, USA) to label proliferating cells, anti-LHR antibodies (bs-6431R, Bioss, USA) to label Leydig cells, or VE-cadherin (sc-28644, SantaCruz, USA) to label vascular endothelium. Tubules with low cell counts and low numbers of proliferating cell nuclear antigen (PCNA)-expressing cells were considered atrophic. Several sections were also stained with hematoxylin and eosin (Dako, USA). Numbers of Sertoli cells and germinal epithelial cells of distinct types were counted on H&E-stained sections based on their distinctive morphologies [21, 22]. Numbers of LHR-positive Leydig cells and vessels were counted on sections using IHC-fluorescence.

### Analysis of serum androgen concentrations

Serum testosterone concentrations were analyzed using an enzyme immunoassay (Hema-Medica, Russia). Blood samples were taken at baseline (before treatment), and at 0, 1, and 3 months after the descent of testes. For each animal, testosterone levels were normalized to their baseline concentrations.

### Isolation of Sertoli and Leydig cells

Sertoli and Leydig cells were isolated from rat testes (mature male Wistar rats) using double enzymatic digestion with further fraction enrichment on a Percoll PLUS (GE Life Sciences, USA) gradient as described previously [23, 24], and were then distinguished according to morphological and immunophenotypic characteristics.

### Manufacture of the Leydig cell secretome

Subconfluent rat Leydig cells at passage 2 were thoroughly washed with Hanks’ solution. Cells were then cultured for 7 days in DMEM-LG and 100 U/ml penicillin/streptomycin at 35 °C. Secretome samples were relieved of cell debris by centrifugation for 10 min at 300*g*. To generate MSC-stimulated Leydig cell secretome samples, Leydig cells were thoroughly washed with Hanks’ solution, incubated in human MSC-conditioned medium for 24 h, washed again, and then cultured at 35 °C for 7 days in DMEM-LG containing 100 U/ml penicillin/streptomycin.

### Sertoli cell migration

Rat Sertoli cells were grown to confluence in 24-well plates containing DMEM-LG supplemented with 2% fetal bovine serum (FBS; Gibco, USA) and 100 U/ml penicillin/streptomycin. Cells were then deprived in basal DMEM-LG for 24 h. Cell monolayers were scratched using a 200-μl pipette tip. After rinsing briefly, cells were treated with basal DMEM-LG as a negative control, human MSC secretome, rat Leydig cell secretome, MSC secretome-stimulated rat Leydig cell secretome, or DMEM-LG + 10% FBS as a positive control. Culture plates were then transferred to an IncuCyte ZOOM Live Cell Analysis System (Essen BioScience, USA) equipped with a × 5 objective. Time-lapse series were continuously acquired every 30 min over 24 h, and 30–50 cells on the edge of the experimental wound were manually tracked. Migration velocities were measured using ImageJ software (USA).

### Statistical analysis

Comparisons between two groups were conducted using the *T* test or Mann-Whitney *U* test. Bonferroni’s correction was used for multiple comparisons. Non-parametric ANOVA with Dunn’s non-parametric many-to-one comparison test was conducted for testosterone level analysis. Chi-squared test was conducted for male rat fertility assessment. Differences were considered significant when *p < 0.05.

## Results

### Local injections of MSC or MSC secretome recovered injured SSC niches

To estimate the regenerative capacity of MSC and MSC secretome, we produced a model of injured SSC niches by imposing bilateral abdominal cryptorchidism on rats. Elevation of testes to the scrotum for 2 weeks led to substantial increases in numbers of atrophic seminiferous tubules during the early stages of niche recovery. Аtrophic tubules had thin germinal epithelial layers and decreased Sertoli cell numbers, indicating substantial germinal cell and SSC niche injury.

**Fig. 1:**
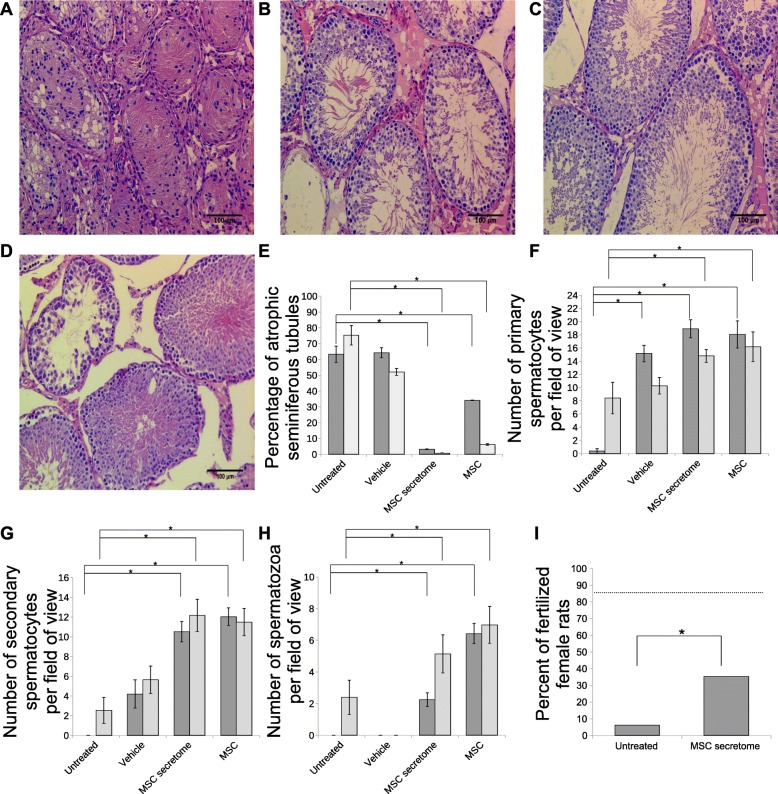
Mesenchymal stromal cell (MSC) secretome injections stimulate recovery of the spermatogonial stem cell (SSC) niche by affecting the whole testicle. H&E-stained testicular tissue sections of the untreated group (**a**), the MSC secretome group (**b**), the unaltered control group (**c**), and the MSC group (**d**); scale bars = 100 μm. Percentages of atrophic seminiferous tubules (**e**); data are presented as means ± standard deviations (SDs) of atrophic seminiferous tubules in three sections from every rat testicle. Quantitative description of the spermatogenic epithelial cell subpopulation. **f** Primary spermatocytes. **g** Secondary spermatocytes. **h** Spermatozoa. Data are presented as mean cell numbers ± standard deviations (SDs) per 40x field of view in three independent sections per testicle. Dark gray bars, 1 month follow-up; bright gray, 3 months. For **e**–**h**, three animals were analyzed for each mean in untreated and MSC groups, and four animals in vehicle and MSC secretome groups. **i** Percentages of female fertilized rats, 16 animals were analyzed in the untreated group, 17 in the MSC secretome group, and 14 in the group of unaltered control animals (dotted line)

We analyzed the effects of local MSC or MSC secretome injections to reveal whether MSCs act in a paracrine manner in SSC niches. We used collagen gel as a carrier to slightly modify the release of MSC secretome components (Additional file 4: Table S2) and prevent their leakage during injections. Local administration of MSC secretome was followed by decreased numbers of atrophic seminiferous tubules and substantial structural and functional recovery of SSC niches (Fig. 1a–e, Additional file 5: Figure S3). Furthermore, spermatogenesis was restored to terminal differentiation forms within 3 months (Fig. 1f–h). The effects of MSCs and their secreted components (MSC secretome) were comparable (Fig. 1e–h). To confirm the functionality of germ cells, male rat fertility restoration was assessed. Preliminary data indicated that MSC secretome injections significantly increased male rat fertility compared to the untreated animals (Fig. 1i).

### Unveiling roles of MSC secretome in coordinated stem cell niche recovery

**Fig. 2:**
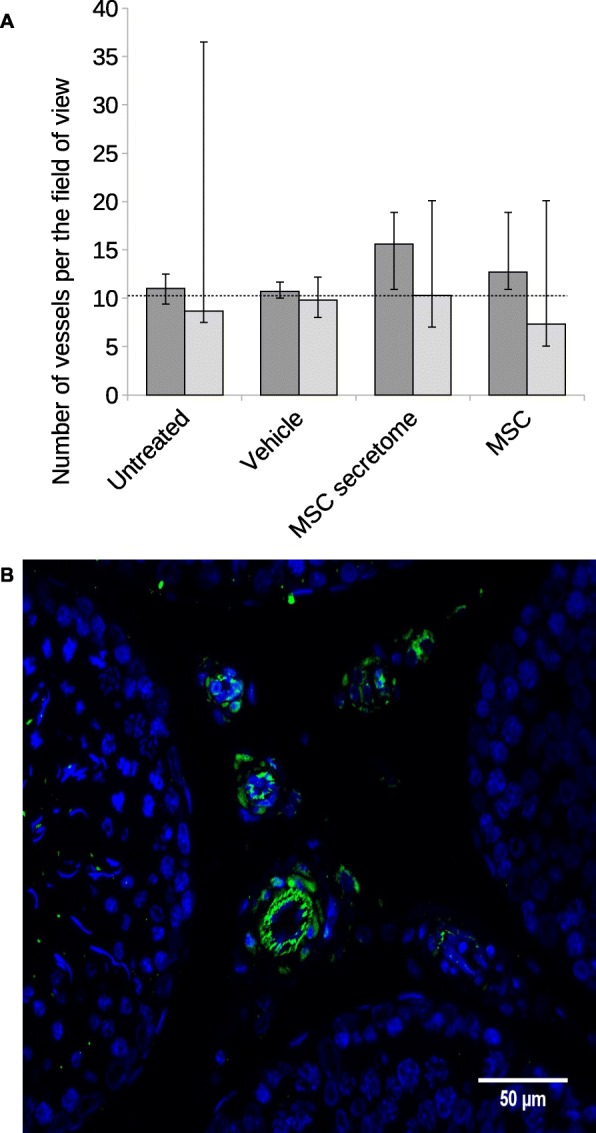
Changes in testicular vascularization following injury. **a** Numbers of vessels per field of view compared with sections from unaltered control animals. Dark gray bars, 1 month follow-up; bright gray, 3 months; dotted line, unaltered control animals, *n* = 2. The results are presented as medians with 25th and 75th percentiles; three sections were analyzed per testicle. Intergroup differences were not significant. Three animals were analyzed for each mean in untreated and MSC groups, and four animals in vehicle and MSC secretome groups. **b** Representative microphotograph of blood vessels on a tissue section; blue pseudocolour, DAPI; green, VE-cadherin; scale bar = 50 μm

To define the ability of MSC secretome to coordinate SSC niche recovery and to elucidate the related mechanisms, we analyzed various niche components. Due to the well-established angiogenic properties of MSC secretome, we determined whether angiogenesis stimulation is important for restoration of the SSC niche to baseline function. We also analyzed changes in vascularization following injury. In these experiments, niche injury did not affect testicular vessel numbers, and these were also unaffected in secretome-injected animals, compared with unaltered control animals (Fig. 2).

**Fig. 3:**
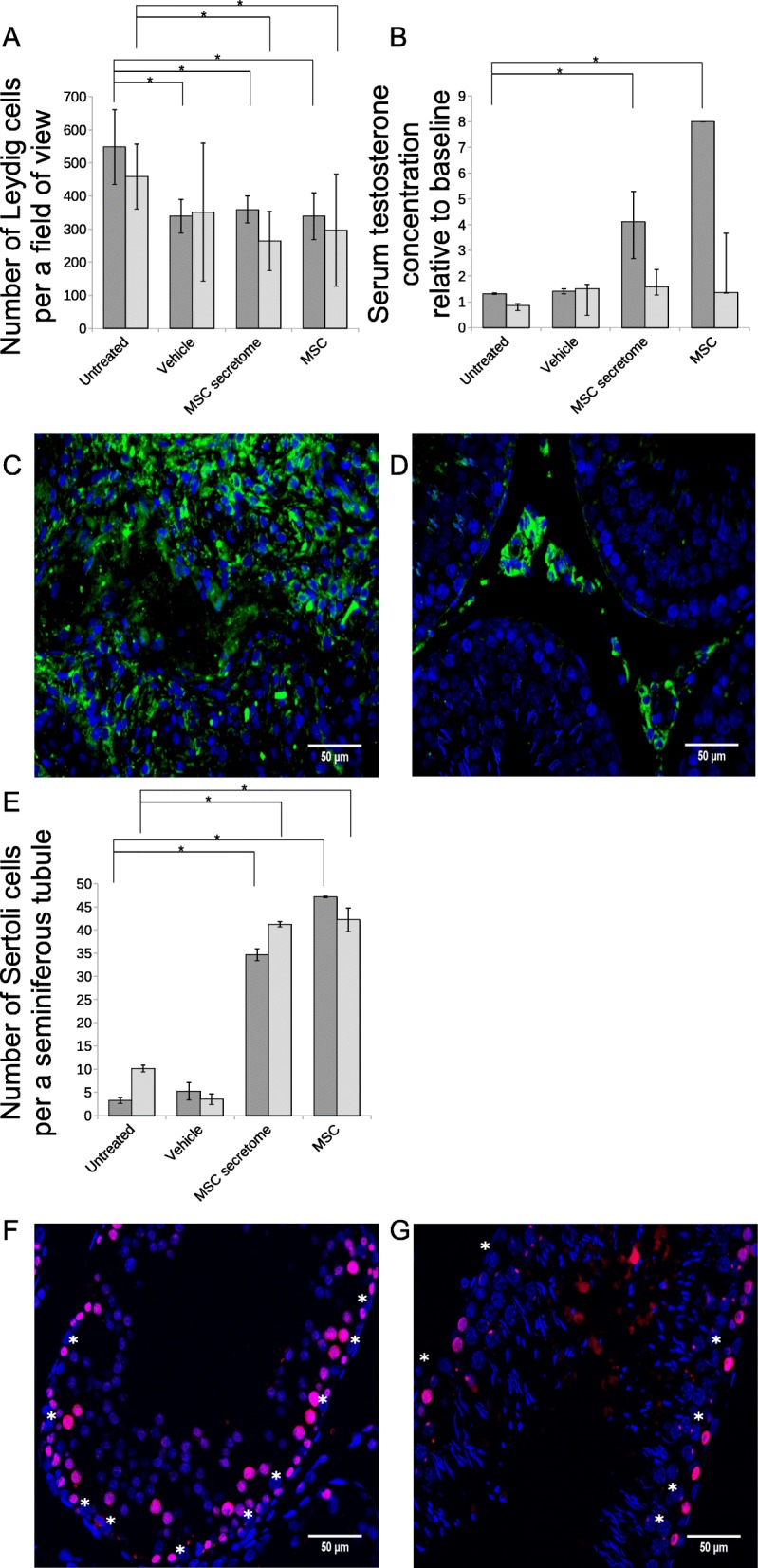
Numbers and functional activities of SSC niche supportive cells. **a** Numbers of interstitial (Leydig) cells per field of view; data are presented as means ± SD of three sections per testicle. Three animals were analyzed for each mean in untreated and MSC groups, and four animals in vehicle and MSC secretome groups. **b** Relative serum testosterone levels normalized to baseline; values are presented as medians with 25th and 75th percentiles. One animal from MSC group with 1 month follow-up, three animals from untreated and 3 months follow-up MSC groups, and four animals from vehicle and MSC secretome groups were sampled for each mean. Dark gray bars, 1 month follow-up; bright gray, 3 months. **c**, **d** Microphotographs of interstitia in tissue sections from untreated animals at 3 months after descent of testes (**c**) and from MSC secretome-treated animals at 3 months after descent of testes (**d**). Blue pseudocolour, DAPI; green, LHR. Scalebars = 50 μm. **e** Numbers of Sertoli cells per seminiferous tubule. Data are presented as means ± SD; three sections were analyzed per testicle. Dark gray bars, 1 month follow-up; bright gray, 3 months. **f**, **g** Microphotographs of seminiferous tubules; MSC secretome-treated animal at 1 month after descent of testes (**f**); MSC secretome-treated animal at 3 months after descent of testes (**g**). Blue pseudocolour, DAPI; magenta, PCNA; scalebars = 50 μm. Asterisks indicate Sertoli cells

Secretory functions of Leydig cells are considered essential for spermatogenesis and fertility. In our functional analyses of Leydig cells, we observed increases in testosterone concentrations in rats within 1 month after MSC secretome injections. Conversely, we found lower testosterone concentrations in untreated animals. Leydig cell numbers were less in MSC secretome-treated rats than in untreated rats. Moreover, Leydig cell numbers and secretory functions decreased towards the 3-month follow-up (Fig. 3a–d).

To confirm recovery of SSC niche as a whole complex after MSC or MSC secretome injections, we analyzed numbers of Sertoli cells, which are known as crucial niche components. Sertoli cell numbers were increased by 1 month after MSC or MSC secretome injections. These effects remained persistent in secretome-treated animals until the end of the experimental period. In contrast, only modest Sertoli cell recovery was observed in untreated animals (Fig. 3).

**Fig. 4:**
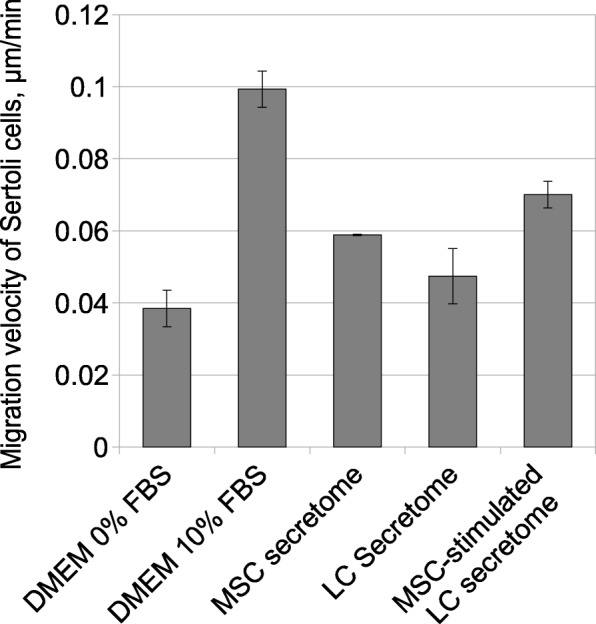
Sertoli cell migration (scratch wound assay)*.* Values are presented as mean velocities in μm/min ± SD of two independent samples per group. Cells were isolated from two animals

In further observations, Sertoli cells did not proliferate at all stages of recovery, suggesting that Sertoli cell pools might be restored via mechanisms other than proliferation (Fig. 3f, g, Additional file 6: Figure S4). Sertoli cell progenitors were previously localized in specific transient zones in adult testis and were considered a possible source for replacement of damaged Sertoli cells in seminiferous tubules [25]. To establish mechanisms that may be involved in recruitment of Sertoli cells, we estimated migration of Sertoli cells following stimulation by Leydig cell secretome, MSC secretome, or MSC-stimulated Leydig cell secretome in vitro. Treatments with MSC secretome stimulated Sertoli cell migration more effectively than treatments with Leydig cell secretome. But secretome samples from MSC-stimulated Leydig cells promoted in vitro migration most strongly (Fig. 4). These data indicate that regenerative effects of the MSC secretome might be realized, at least in part, by activation of SSC niche components followed by a complex of recovery processes in testis.

## Discussion

Adult stem cells within stem cell niches are likely core participants in tissue regeneration and homeostasis. Yet the mechanisms by which niche restoration is managed after tissue injury remain elusive. Among components that participate in the recovery of stem cell niches, MSCs play key roles in supporting and maintaining stem cells under physiological conditions and after tissue injury. Thus, using the SSC niche as a model, we investigated MSC regulatory functions in stem cell niches.

To analyze the potency of MSC secretome to stimulate recovery of spermatogenesis, we injected the mixture of MSC secretome with collagen gel. Collagen is one of the most investigated natural polymers for tissue engineering scaffolds, and its ability for inducing regeneration processes with delivered growth factors has been well established. The advantages of collagen materials also include biocompatibility, degradability and biomimetic chemical properties, the absence of toxic properties, weak immunogenicity, and high mechanical strength [26–28].

We demonstrate herein that MSC secretome stimulates recovery of spermatogenesis with comparable potency to MSCs themselves. In the present study, numbers of primary spermatocytes as well as numbers of Leydig cells were also comparable in secretome-, MSC-, and vehicle-treated animals at 1 month after injection. This might be due to the ability of the individual components of DMEM-LG to support high metabolic demands of Sertoli and germ cells at initial stages of recovery [29]. Proliferation of Leydig cells may have been inhibited by germ cells [30] in which numbers were reportedly increased in vehicle-treated rats [31]. However, spermatogenesis remained dysfunctional in the vehicle group. Therefore, the nutritional effects were not sufficient for recovery of functional spermatogenesis, warranting further studies of role of the MSC secretome in spermatogonial stem cell niche recovery.

Because disruption of blood supply is considered a major cause of failed spermatogenesis, we determined blood vessel numbers and areas in the present rat testicles. Contrary to several publications [32, 33], the angiogenic potential of MSCs, mostly mediated by secreted angiogenic factors, did not influence the recovery of spermatogenesis. These results might be associated with the absence of changes in vessel numbers in testicular interstitia of this injury model.

Three months after injections of MSCs or MSC secretome samples, germ cells were differentiated to terminal forms. To maintain the development of primary spermatocytes and their progeny, Sertoli cell barriers that form immune-privileged zones and other specific microenvironments need to be formed first. Intensive spermatogonial apoptosis was previously considered an indirect physiological response to excess numbers of cells produced by spermatogonial proliferation [34]. More recently, spermatogonial apoptosis was associated with maturity of Sertoli cell barriers in seminiferous tubules [35]. Herein, injections of MSC secretome were followed by the formation of Sertoli cell barriers during the early stages of recovery. These may provide a supportive environment for further recovery over our 3-month observation period.

MSCs may mimic Sertoli cell functions and contribute to the early stages of spermatogonial lineage expansions by supporting spermatogonia pools through secretions of GDNF, FGF2, and other paracrine factors that are important for spermatogenesis [36]. MSCs have been identified as substitute supportive cells in other injured stem cell niches, such as those of the intestine, where Gli1-expressing MSC provided Wnt for stem cells and transiently executed epithelial Paneth cell functions [12]. We assume that similar processes occur in SSC niches. Recently, it was shown that co-transplantation of SSC with MSC significantly improved the recovery of endogenous SSC and increased the homing efficiency of transplanted SSC [37]. Although these observations confirm the supportive functions of MSC, Sertoli cells are indispensable for normal spermatogenesis, and locally injected MSC or MSC secretome cannot replace all Sertoli cell functions. Because Sertoli cells are terminally differentiated and have limited proliferation potential, we suggest that other mechanisms of Sertoli cell pool restoration are active in SSC niches. The transition region between rete testis and seminiferous tubules contains undifferentiated progenitors of Sertoli cells, which can proliferate [25]. Hence, Sertoli cells likely migrate to regions of seminiferous tubules upon restoration of spermatogenesis. Although few studies show migration of Sertoli cells from transitional regions to seminiferous tubules, our results indirectly support this hypothesis, because we did not observe proliferating Sertoli cells in restorative tubules and additionally demonstrated that the MSC secretome stimulates Sertoli cell migration in vitro.

In the present experiments, injected MSC and MSC secretome stimulated secretory functions of Leydig cells. But we did not define the targets of MSC secretory molecules among analyzed SSC niche components. Whereas Leydig cells maintained Sertoli cell functions and population numbers, Sertoli cells were previously shown to positively regulate Leydig cell testosterone production, and these effects were not species specific [38, 39].

Several investigators have shown similarities between Sertoli cells and MSC [40], although MSC did not originate from Sertoli cells, at least not in in vitro experiments [41]. Therefore, MSC might support or attract stem cell niche components and/or mimic the paracrine signals of absent niche cells. Accordingly, insulin-like growth factor (IGF) was present in MSC secretome and promoted testosterone production by Leydig cells in other studies [38, 42]. This hypothesis is consistent with an advanced conception of the regenerative potential of MSC [43], for which consideration as effectors has been replaced with consideration as regulators that transiently provide paracrine stimuli for target cells and trigger regenerative processes in tissues after damage. These roles of MSC in stem cell niches should be further investigated.

In conclusion, our results demonstrate that the MSC secretome stimulates SSC niche recovery. Given the comparable effects of MSC and MSC secretome, a paracrine function of MSC is evident. Moreover, niche recovery may be supported by MSC-mediated restoration of critical SSC niche components, including Leydig and Sertoli cells. Conceivably, MSCs support or mimic components of stem cell niches and concomitantly attract missing components to speed recovery. Taken together, our data suggest a coordinating role of MSC during stem cell niche recovery and indicate the importance of further research in this field.

## Conclusions

In conclusion, the authors examined the roles of MSC with focus on their secretome in stem cell niche recovery using spermatogonial stem cell (SSC) niche as a model. Local subtunical injections of MSC or MSC secretome were sufficient to recover spermatogenesis and production of functional germ cells. Possibly, MSCs mimic the functions of lost supportive cells within the stem cell niche triggering tissue regenerative processes after damage. Further research in this field is required to translate this strategy in regenerative medicine.

## Supplementary information

**Additional file 1: Figure S1.** Phenotypic characterization of adipose-derived MSC.

**Additional file 2: Table S1.** Threshold levels of selected growth factor concentrations in MSC secretome samples measured by ELISA.

**Additional file 3: Figure S2.** Transmission electron microscopy images of extracellular vesicles obtained from MSC secretome samples.

**Additional file 4: Table S2.** Retention of growth factors in collagen gel 4 hours after its subcutaneous administration to experimental animals.

**Additional file 5: Figure S3.** Microphotographs of testicular tissue sections.

**Additional file 6: Figure S4.** Microphotographs of seminiferous tubules.

## Data Availability

All data generated and/or analyzed during this study are available from the corresponding author upon reasonable request.

## Abbreviations

DAPI: 4′,6-Diamidino-2-phenylindole
DMEM: Dulbecco’s modified Eagle’s medium
FBS: Fetal bovine serum
FGF2: Basic fibroblast growth factor
GDNF: Glial cell line-derived neurotrophic factor
H&E: Hematoxylin and eosin
IGF: Insulin-like growth factor
IHC: Immunohistochemistry
LHR: Luteinizing hormone/choriogonadotropin receptor
MSCs: Mesenchymal stromal cells
MWCO: Molecular weight cutoff
PCNA: Proliferating cell nuclear antigen
SD: Standard deviation
SSCs: Spermatogonial stem cells
