# Supplementary figures and images for: A magic kick for regeneration: role of mesenchymal stromal cell secretome in spermatogonial stem cell niche recovery
Source: Stem Cell Res Ther. 2019 Nov 21;10:342. doi: 10.1186/s13287-019-1479-3 (PMC6873442; doi:10.1186/s13287-019-1479-3)

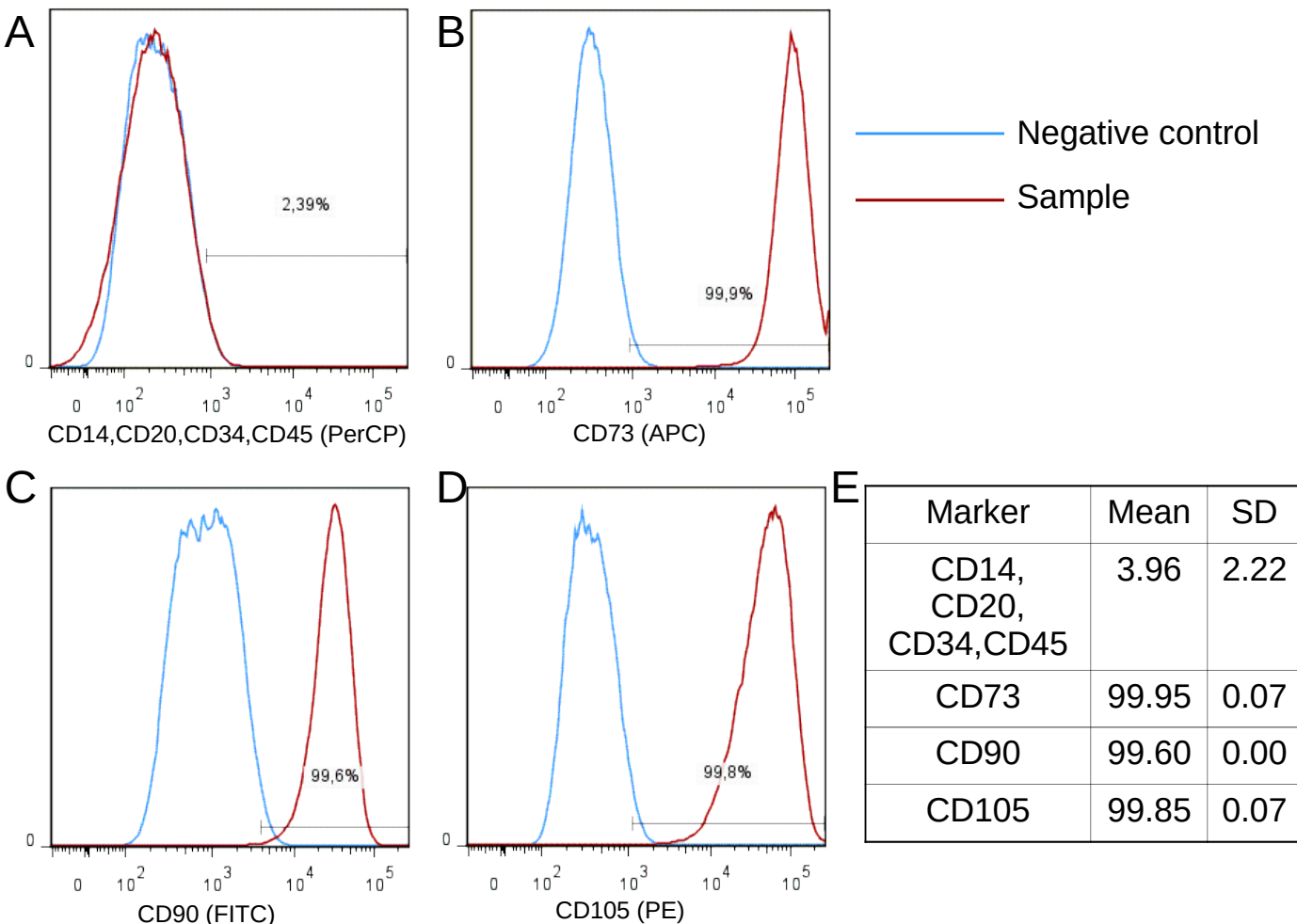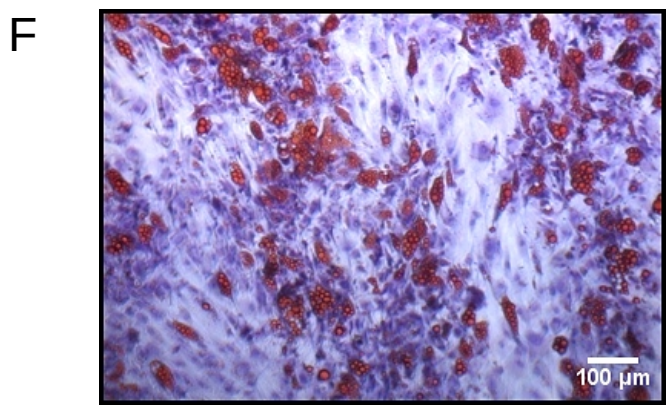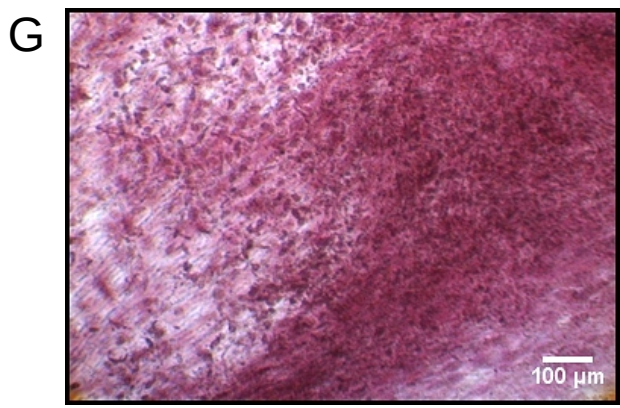

Supplement: Supplementary file 1 — Additional file 1: Figure S1. Phenotypic characterization of adipose-derived MSC. [file 13287_2019_1479_MOESM1_ESM.pdf]

A

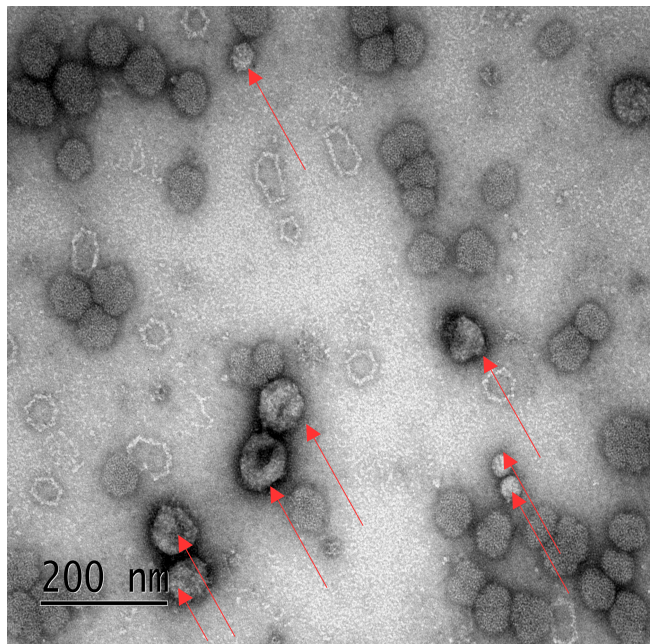

B

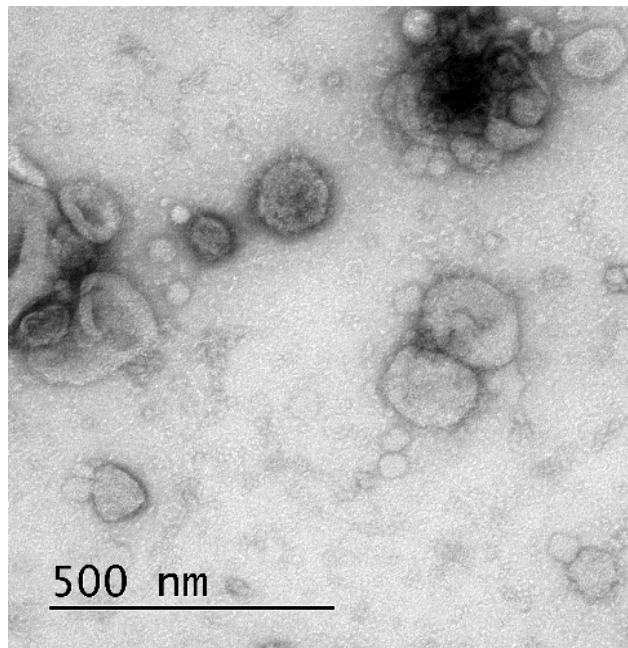

Supplement: Supplementary file 3 — Additional file 3: Figure S2. Transmission electron microscopy images of extracellular vesicles obtained from MSC secretome samples. [file 13287_2019_1479_MOESM3_ESM.pdf]

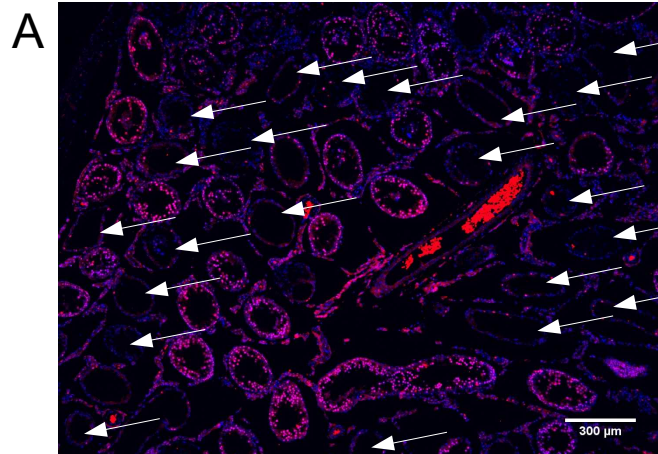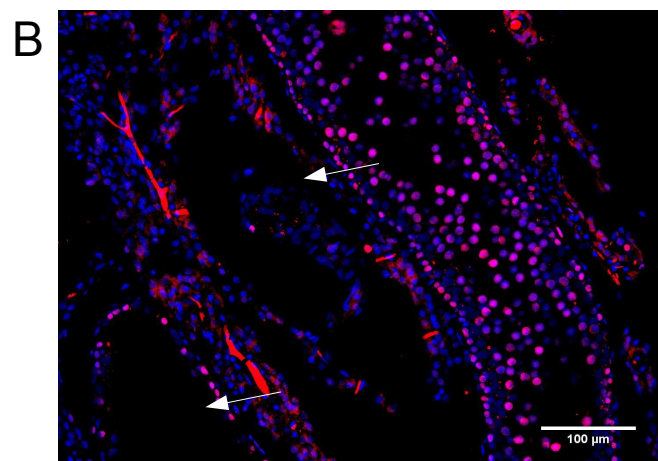

Supplement: Supplementary file 5 — Additional file 5: Figure S3. Microphotographs of testicular tissue sections. [file 13287_2019_1479_MOESM5_ESM.pdf]

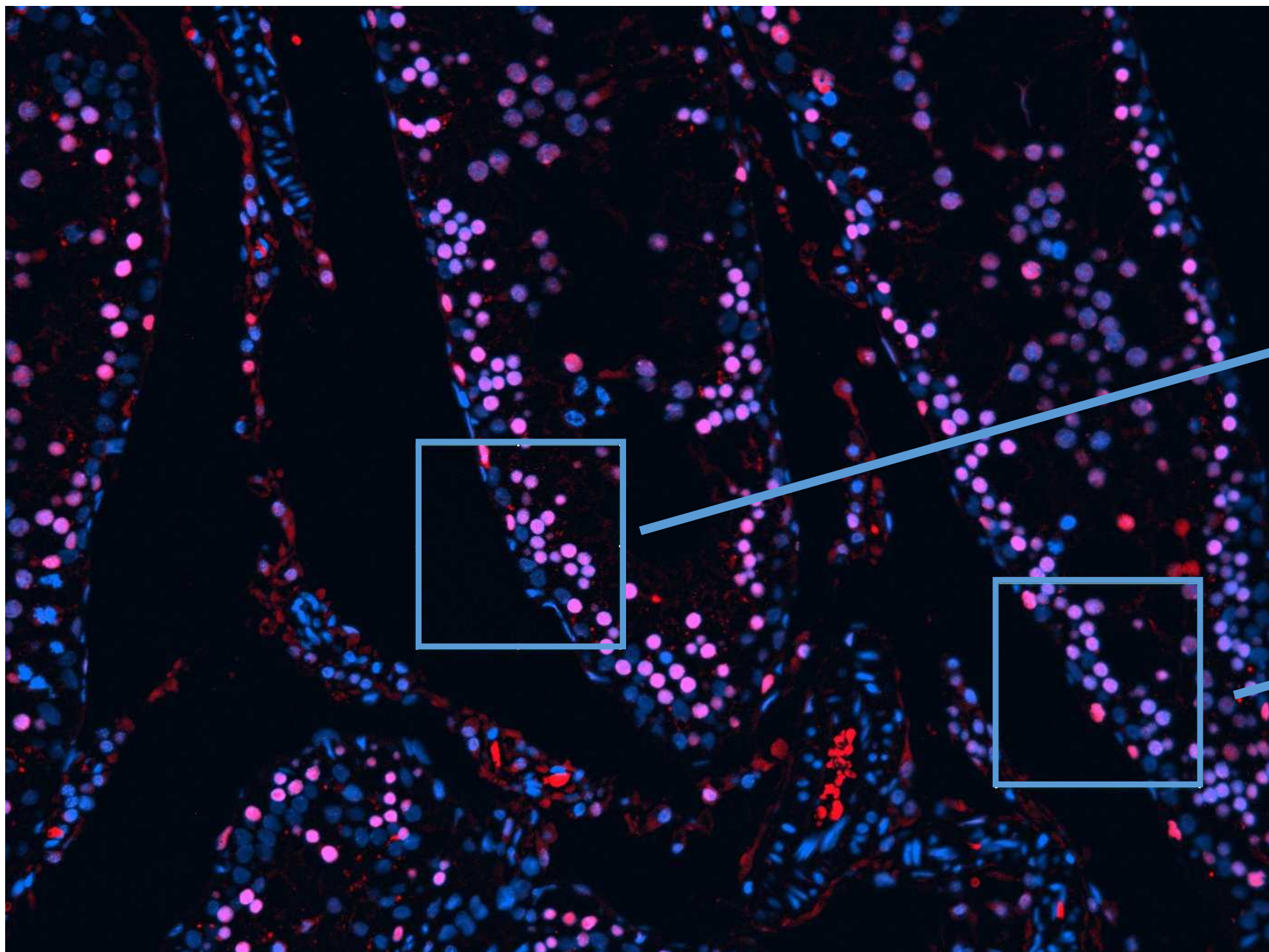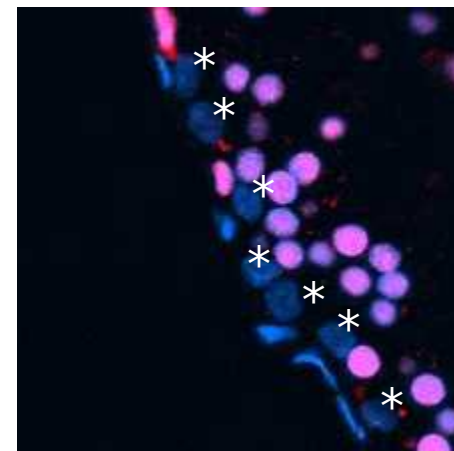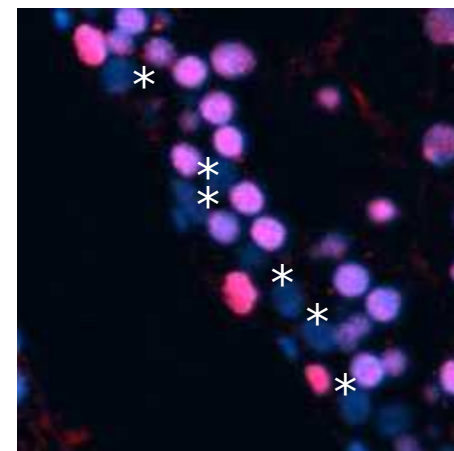

Supplement: Supplementary file 6 — Additional file 6: Figure S4. Microphotographs of seminiferous tubules. [file 13287_2019_1479_MOESM6_ESM.pdf]
