# Supplementary material for: A magic kick for regeneration: role of mesenchymal stromal cell secretome in spermatogonial stem cell niche recovery
Source: Stem Cell Res Ther. 2019 Nov 21;10:342. doi: 10.1186/s13287-019-1479-3 (PMC6873442; doi:10.1186/s13287-019-1479-3)
Supplement: Supplementary file 2 — Additional file 2: Table S1. Threshold levels of selected growth factor concentrations in MSC secretome samples measured by ELISA. [file 13287_2019_1479_MOESM2_ESM.docx]

| Factor | VEGF | HGF | FGF2 | Angpt-1 | PEDF | GDNF |
| --- | --- | --- | --- | --- | --- | --- |
| Minimal threshold concentration, pg/ml (5th percentile) | 200 | 150 | 0.29 | 45 | 500 | 44 |

**Table S1. Threshold levels of selected growth factor concentrations in MSC secretome samples measured by ELISA.**

Due to complex composition of MSC-CM, it was necessary to focus on components that would reflect its regenerative potency for a specific condition. In our previous study, we selected several factors crucial for MSC secretome-mediated tissue regeneration.
