# Supplementary material for: A magic kick for regeneration: role of mesenchymal stromal cell secretome in spermatogonial stem cell niche recovery
Source: Stem Cell Res Ther. 2019 Nov 21;10:342. doi: 10.1186/s13287-019-1479-3 (PMC6873442; doi:10.1186/s13287-019-1479-3)
Supplement: Supplementary file 4 — Additional file 4: Table S2. Retention of growth factors in collagen gel 4 hours after its subcutaneous administration to experimental animals. [file 13287_2019_1479_MOESM4_ESM.docx]

| Growth factor | Retention, % |
| --- | --- |
| VEGF | 18 |
| HGF | 45 |

**Table S2. Retention of growth factors in collagen gel 4 hours after its subcutaneous administration to experimental animals.**

The means from 2 rats are presented in the table. To perform this experiment, a mixture of collagen gel with the MSC secretome with known concentrations of the growth factors was polymerized at 37^o^C for 30 min. Then anesthetized rats were administered polymerized samples subcutaneously. After 4 hours, the samples were isolated and analyzed using an ELISA.
